# Human seminal plasma suppresses neutrophil antimicrobial functions and promotes bacterial survival

**DOI:** 10.3389/fimmu.2026.1846703

**Published:** 2026-07-14

**Authors:** Gabriel Mayoral-Andrade, Gabriela Vásquez-Martínez, Israel Cotzomi-Ortega, Gamaliel Sánchez-Orellana, Jeimy M. Lopez-Torres, Diana Zepeda-Orozco, Laura Pérez-Campos Mayoral, María Teresa Hernández-Huerta, Abraham Salvador Majluf-Cruz, María del Socorro Pina-Canseco, Margarito Martínez-Cruz, Gabriel Mayoral-Canseco, Carlos Alejandro Vásquez-Martínez, Víctor Cruz Hernández, Luz María Moreno Pombo, Adriana Carriedo Blanco, Eduardo Pérez-Campos Mayoral, Héctor Martínez-Ruiz, Eduardo Pérez-Campos, Juan de Dios Ruiz-Rosado

**Affiliations:** 1 The Kidney and Urinary Tract Research Center, Abigail Wexner Research Institute, Nationwide Children’s Hospital, Columbus, OH, United States; 2 Department of Pediatrics, Ohio State University, Columbus, OH, United States; 3 Center for Research Faculty of Medicine UNAM-UABJO, Faculty of Medicine and Surgery, Autonomous University Benito Juárez of Oaxaca, Oaxaca, Mexico; 4 Division of Pediatric Nephrology and Hypertension, Nationwide Children’s Hospital, Columbus, OH, United States; 5 SECIHTI, Faculty of Medicine and Surgery, Autonomous University Benito Juárez of Oaxaca, Oaxaca, Mexico; 6 Regional General Hospital 1 “Dr. Carlos MacGregor Sánchez Navarro”, Medical Research Unit in Thrombosis, Hemostasis, and Atherogenesis, Mexico City, Mexico; 7 Biochemistry and Molecular Biology, National Technological of Mexico/IT Oaxaca, Oaxaca, Mexico; 8 Hospital General Dr. Aurelio Valdivieso, IMSS-Bienestar, Oaxaca, Mexico; 9 Clinical Pathology Laboratory “Dr. Eduardo Pérez Ortega”, Oaxaca, Mexico

**Keywords:** bacterial clearance, human seminal plasma, NETosis, neutrophils, phagocytosis, prostaglandins, reactive oxygen species, uropathogenic *E. coli* (UPEC)

## Abstract

Human seminal plasma (HSP) plays an important role in shaping the reproductive immune environment, but its effects on modulating neutrophil antimicrobial functions and pathogen clearance remain unclear. In this study, we investigated the immunomodulatory effects of HSP on key neutrophil effector responses, including reactive oxygen species (ROS) production, neutrophil extracellular trap (NET) formation, phagocytosis, and bacterial killing. Human neutrophils were stimulated with calcium ionophore A23187, phorbol 12-myristate 13-acetate (PMA), or uropathogenic *Escherichia coli* (UPEC) in the presence or absence of HSP. In addition, neutrophils from NOX2- and PAD4-knockout mice were used to elucidate the molecular pathways underlying HSP-mediated regulation of NETosis. HSP significantly suppressed ROS production and NET formation induced by A23187, PMA, and UPEC, while also reducing neutrophil phagocytic capacity and impairing bacterial killing. Mechanistically, HSP-mediated inhibition of NETosis was found to be PAD4-dependent but NOX2-independent. Furthermore, the inhibitory effect of HSP on PMA-stimulated human neutrophils was diminished when HSP was obtained from donors pretreated with acetylsalicylic acid, which significantly reduced prostaglandin E2 levels in HSP. Consistent with this observation, pharmacological blockade of prostaglandin signaling restored ROS production in HSP-treated neutrophils. Overall, these findings identify HSP as a physiological inhibitor of neutrophil effector functions and support a role for HSP in maintaining immune homeostasis and tolerance within the reproductive tract through the suppression of neutrophil oxidative burst, NET formation, and antimicrobial activity.

## 1 Introduction

Seminal plasma contributes to the establishment of maternal immune tolerance to paternal alloantigens through the induction of regulatory pathways, including the differentiation of tolerogenic dendritic cells and CD4^+^CD25^+^Foxp3^+^ regulatory T cells (Tregs), while also regulating cytotoxic natural killer cell and neutrophil responses, processes that support embryo implantation (1–6).

Despite its tolerogenic properties, exposure to semen is also associated with a transient inflammatory response in the female reproductive tract. Studies in murine and livestock models indicate that seminal fluid components and spermatozoa can induce rapid, transient accumulation of neutrophils or granulocytes within the uterus after mating or insemination (7–11). This response is context-dependent and may vary by species, hormonal status, microbiota, and infection (7, 12, 13). Within this complex mucosal environment, neutrophils may contribute to reproductive tract homeostasis by clearing pathogens, cellular debris, excess spermatozoa, and seminal plasma components, thereby helping to maintain tissue integrity and limit microbial invasion.

Neutrophils are central mediators of innate immunity. Upon recognition of pathogen-associated molecular patterns (PAMPs), neutrophils deploy multiple antimicrobial effector mechanisms, including phagocytosis, reactive oxygen species (ROS) production, degranulation, and the formation of neutrophil extracellular traps (NETs) (14). These structures are composed of decondensed DNA (15, 16), generated by activation of peptidyl arginine deiminase 4 (PAD4), together with post-translationally modified proteins (17–20). Because NETs formation may be accompanied by cell death, this process is referred to as NETosis (20–23). In both human and murine models, NETosis can be triggered by reactive oxygen species (ROS) (24–27) produced by the NADPH oxidase (NOX2) complex (28–30).

Increasing evidence indicates that NETosis can affect reproductive health. Spermatozoa can induce NETosis, leading to sperm entrapment and a significant reduction in motility (31, 32). The interaction between neutrophils and sperm, as well as the activation of adverse immune responses in the female reproductive tract, is largely modulated by HSP. This fluid contains multiple immunosuppressive factors, such as prostaglandins (PGs), that regulate the local immune response during fertilization and early embryonic development (33, 34). HSP has been reported to inhibit key processes in monocytes and granulocytes, such as phagocytosis and oxidative burst (35–37). However, the mechanisms by which HSP regulates these effector functions, and the implications for antimicrobial host defense, remain poorly understood.

In the present study, we show that HSP exerts a marked immunomodulatory influence on neutrophil effector responses. Exposure to HSP attenuated neutrophil effector functions, including ROS production, phagocytosis, and NET formation, in response to both inflammatory and infectious stimuli, resulting in impaired clearance of uropathogenic *E. coli*. Mechanistically, HSP-mediated suppression of NETosis was found to be PAD4-dependent but NOX2-independent. Additionally, our findings implicate prostaglandin E2 signaling as a potential mediator of the reduced neutrophil oxidative burst observed following HSP exposure.

Collectively, these findings establish HSP as a physiological regulator of neutrophil effector functions and delineate distinct mechanisms through which it suppresses ROS production and NET formation, thereby attenuating neutrophil-mediated antibacterial activity.

## 2 Materials and methods

### 2.1 Study design and experimental procedures

Human blood from healthy individuals was obtained through an anonymized donor program (IRB14-00376) approved by The Nationwide Children’s Hospital Institutional Review Board. The animal protocol in this study was reviewed and approved by the Institutional Animal Care and Use Committee at the Abigail Wexner Research Institute at Nationwide Children’s Hospital (Protocol Number AR13-00057). Isolated human and murine neutrophils were stimulated with the calcium ionophore A23187, phorbol 12-myristate 13-acetate (PMA)(Sigma-Aldrich, Merck KGaA, Darmstadt, Germany), or uropathogenic *Escherichia coli* (UPEC, strain CFT073) at multiplicities of infection (MOI) of 10 or 100 to simulate a moderate or a severe infection, respectively, based on our previous publications (38, 39). ROS production, bactericidal activity against UPEC, phagocytic capacity, and NET formation were quantified as outlined below. These parameters were assessed in neutrophils pre-incubated with HSP or media alone. HSP was obtained from healthy donors and from individuals treated with acetylsalicylic acid (ASA; 500 mg orally every 8 h for 3 days) prior to semen donation, as previously described by our group (40). The HSP was diluted 1:10 in phosphate-buffered saline (PBS; pH 7.4) to ensure a consistent effect across all samples. This prospective experimental study was approved by the Ethics and Research Committee of the Dr. Aurelio Valdiveso General Hospital, Oaxaca, Mexico.

### 2.2 Neutrophil isolation

Human neutrophils were isolated from peripheral blood of healthy donors using the EasySep™ Direct Human Neutrophil Isolation Kit (STEMCELL Technologies Canada Inc), according to the manufacturer’s instructions (41). Murine neutrophils were isolated from femurs and tibias using the MojoSort™ Mouse Neutrophil Isolation Kit (BioLegend, United States) according to the manufacturer’s instructions.

#### 2.2.1 Neutrophil viability

Neutrophils undergoing apoptosis were evaluated using the Pacific Blue Annexin-V Apoptosis Detection Kit with 7-Aminoactinomycin D (7-AAD; Biolegend, United States). Neutrophils were acquired on an LSR II cytometer (BD Biosciences, United States), and data were analyzed using FlowJo software (v10.8.2).

### 2.3 Seminal plasma collection

All subjects who donated seminal plasma had previously signed informed consent forms. Healthy donors underwent a semen analysis study within the reference range according to World Health Organization criteria (42, 43), performed by the Clinical Pathology Laboratory “Dr. Eduardo Pérez Ortega. Two groups, each consisting of five patients, were established. In the first group, donors received 500 mg of acetylsalicylic acid (ASA) every 8 hours for three days prior to semen collection, generating HSP from ASA-treated donors (HSP-ASA). ASA was administered to reduce the levels of cyclooxygenase-dependent proteins, including prostaglandins, in seminal plasma. Donors in the second group did not receive ASA treatment and served as untreated controls, providing human seminal plasma (HSP). Semen samples were collected after up to 3 days of sexual abstinence and were evaluated for volume, pH, sperm concentration, and motility. Human seminal plasma (HSP) was then subjected to two consecutive centrifugation steps at 6,500 rpm for 5 minutes each (43, 44).

#### 2.3.1 Quantification of prostaglandin E_2_ and total protein in human seminal plasma

For PGE_2_ and total protein quantification, seminal plasma samples (n = 5, HSP and HSP-ASA) were analyzed. PGE_2_ concentrations were measured using a Prostaglandin E_2_ Express ELISA kit (Cayman Chemical, United States), following the manufacturer’s instructions. Total protein concentration was quantified using the Pierce™ Dilution-Free™ Rapid Gold BCA Protein Assay Kit (Thermo Fisher Scientific, United States), according to the manufacturer’s protocol.

### 2.4 Quantification of ROS by DHE oxidation

Mouse or human neutrophils (2 × 10^5^ cells) were resuspended in 200 μL of RPMI 1640 medium (Life Technologies Corporation, United States) supplemented with 10% fetal bovine serum (FBS; Life Technologies Corporation, United States) and seeded into black-walled, clear-bottom 96-well plates (Corning Incorporated - Life Sciences, United States). Neutrophils were pre-incubated in the presence or absence of HSP for 30 min at 37 °C and then stimulated with 100 μM calcium ionophore A23187 (Sigma-Aldrich, Germany), 50 nM PMA (Abcam, United States), or incubated with UPEC (strain CFT073) (45) at a multiplicity of infection (MOI) of 10 or 100. For UPEC stimulation, bacteria were grown under standardized culture conditions, and bacterial concentrations were estimated by optical density measurements and confirmed by colony-forming unit (CFU) enumeration. The bacterial inoculum was then adjusted according to the number of neutrophils seeded per well (2 × 10^5^ cells) to achieve the desired MOI, corresponding to 2 × 10^6^ bacteria/well for MOI 10 and 2 × 10^7^ bacteria/well for MOI 100. ROS production was assessed by measuring the oxidation of dihydroethidium (DHE; Invitrogen, United States). The concentrations of A23187 and PMA, as well as the bacterial MOI, were selected based on published NETosis and ROS assays demonstrating consistent induction of measurable ROS and NET formation (38, 46, 47). Fluorescence kinetics were recorded for 2 h at 37 °C using a SpectraMax M2 microplate reader (Molecular Devices). To determine whether the effect of HSP on ROS production in human neutrophils is mediated by prostaglandin signaling, neutrophils were pre-incubated for 30 minutes with 1 µM of each inhibitor in a cocktail containing three prostaglandin antagonists before incubation with HSP. This cocktail includes receptor antagonists for IP (prostacyclin, Cayman Chemical, United States), EP4 (Cayman Chemical, United States), and EP2 receptors (Cayman Chemical, United States). These concentrations of prostaglandin antagonists were selected based on published assays demonstrating consistent inhibition of prostaglandin signaling (48).

### 2.5 Visualization and quantification of NETs

Human or murine neutrophils (2 × 10^5^ cells) were seeded into NEST Scientific 8-wells (Stellar Scientific, United States) and pre-incubated with or without HSP for 30 minutes at 37 °C. Cells were then stimulated with 100 μM of calcium ionophore A23187, 50 nM of PMA, or UPEC, strain CFT073 at MOI 100 for 3 hours at 37 °C. Neutrophils were fixed overnight with 4% PFA (Sigma-Aldrich, Germany). Following two PBS washes, cells were stained overnight at 4 °C with anti-myeloperoxidase (MPO) antibody (1:200, Abcam, United States) and anti-dsDNA antibody (1:200, Abcam, United States) to visualize NETs (49). Secondary antibodies Alexa Fluor 568 (1:1000 Abcam, United States) and Alexa Fluor 488 (1:1000, Abcam, United States) were added for 1 hour at room temperature. Nuclear staining was performed using NucBlue™ reagent (Invitrogen, United States). Fluorescent imaging was conducted using an EVOS^®^ FL microscope (Life Technologies Corporation, United States) (50). NETs were analyzed using a Fiji (ImageJ) algorithm (51).

### 2.6 Phagocytosis assay

Human neutrophils (2 × 10^5^ cells/well) were seeded in black-walled, clear-bottom 96-well plates (Corning Incorporated - Life Sciences, United States). After 30 minutes of incubation with HSP or media, cells were stimulated with fluorescein-labeled LPS-coated bioparticles (FITC-Beads; Vybrant™ Phagocytosis Assay Kit, Life Technologies Corporation, United States) for 5, 15, and 30 minutes (52). Neutrophils were subsequently fixed overnight with 4% paraformaldehyde (PFA). Cells were stained with 0.25 mg/mL trypan blue (Gibco, United States) and imaged using the EVOS^®^ FL microscope. Four fields per well were analyzed to quantify the number of phagocytic cells and their mean fluorescence intensity (MFI). Image analysis was performed using Fiji software.

### 2.7 UPEC killing

UPEC killing assays were performed as previously described (38). Briefly, human neutrophils were seeded in 96-well plates and pre-incubated in the presence or absence of HSP for 30 min. Neutrophils were then infected with UPEC (strain CFT073) at MOI of 10 or 100 and incubated at 37 °C. At 45 min post-infection, neutrophils were centrifuged at 500 × g for 5 min. Supernatants (10 μL) were collected to determine the extracellular bacterial burden, and the remaining supernatants were discarded. Cells were then treated with gentamicin (5mg/mL; Life Technologies Corporation, United States) for 10 min to eliminate extracellular bacteria. Neutrophils harboring intracellular bacteria were centrifuged and washed twice with PBS, resuspended in RPMI 1640 medium supplemented with FBS, and incubated for an additional 3 h at 37 °C to allow neutrophil-mediated elimination of UPEC. After incubation, neutrophils were lysed with PBS containing 0.1% Triton X-100 (Fisher Scientific, United States) to determine the intracellular bacterial burden. Extracellular and intracellular UPEC were plated on LB agar plates (Fisher Scientific, United States) and incubated overnight at 37 °C. Bacterial burden was quantified by colony-forming unit (CFU) counting.

### 2.8 Animals

Males and females, 8–10 weeks, NOX2-KO mice, (B6.129S-Cybb^tm1Din^/J) (*Cybb^-/-^*) and PAD4-KO, (B6.Cg-Padi4^tm1.1Kmow^/J) (*Padi4^-/-^*) were obtained from The Jackson Laboratory and housed under controlled environmental conditions with free access to food and water (53).

### 2.9 Statistical analysis

Statistical analyses were performed using GraphPad Prism 9 (GraphPad Software, La Jolla, CA, USA). No data were excluded from the analyses. Data distribution was assessed using the Shapiro–Wilk test for normality. Comparisons between two groups were performed using an unpaired Student’s *t*-test for normally distributed data. Paired data were analyzed using a paired Student’s *t*-test when normally distributed. Comparisons among multiple groups were analyzed using one-way ANOVA followed by Tukey’s *post hoc* test or two-way ANOVA with Tukey’s or Šídák’s multiple comparisons test for normally distributed data. Unless otherwise indicated, experiments included at least three technical replicates per condition and were repeated in at least two independent experiments. Data are presented as mean ± SD. Statistical significance was defined as *P* ≤ 0.05.

## 3 Results

### 3.1 HSP negatively regulates NET formation and antimicrobial function in human neutrophils

**Figure 1:**
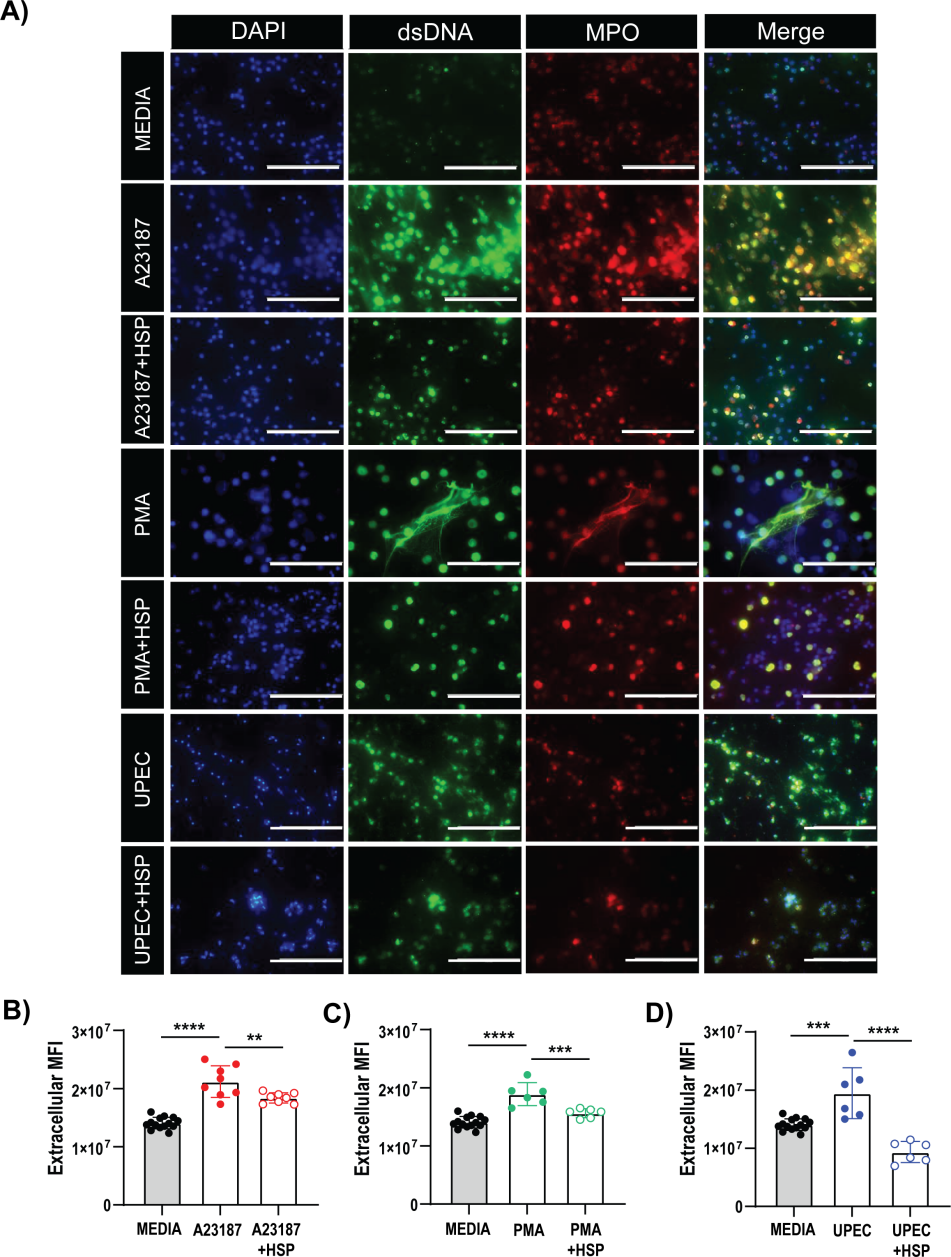
HSP inhibits NETosis in human neutrophils stimulated with PMA, A23187, and UPEC. **(A)** Representative microscopy images of human neutrophils undergoing NETosis, three hours after stimulation with Uropathogenic *Escherichia coli* (UPEC), Phorbol myristate acetate (PMA), or calcium ionophore A23185, alone or in combination with human seminal plasma (HSP). Stimulated neutrophils were stained for double-stranded deoxyribonucleic acid (dsDNA, green), myeloperoxidase (MPO, red), and NucBlue™ (blue). Images were captured at 40x magnification. Scale bar = 100 µm. **(B-D)** Bar graphs showing the mean fluorescence intensity (MFI) of extracellular DNA release from human neutrophils, pre-incubated with media or HSP, undergoing NETosis following exposure to **(B)** A23187, **(C)** PMA, and **(D)** UPEC at a multiplicity of infection (MOI) of 100. All data are presented as mean ± SD. Data were obtained from 2 independent experiments, with *n* = 6–8 per group. Normality was assessed using the Shapiro–Wilk test. Statistical significance was determined by one-way ANOVA followed by Tukey’s multiple-comparisons test. p < 0.01**, p < 0.001***, and p < 0.0001****.

We first sought to investigate whether HSP modulates the effector functions of activated human neutrophils. To this end, isolated human neutrophils were pre-incubated with HSP or media (control) and then stimulated with the calcium ionophore A23187 or PMA, two well-established inducers of NETs formation. Stimulation with A23187 or PMA effectively induced NETosis in neutrophils, as evidenced by the release of extracellular DNA enriched with myeloperoxidase (MPO) in cells preincubated with media alone (control, **Figures 1A–C**). In contrast, pre-incubation with HSP significantly reduced NET formation in response to both A23187 (*p =* 0.0047; **Figure 1B**) and PMA (*p =* 0.0004; **Figure 1C**). These results indicate that HSP significantly suppresses NET release in activated human neutrophils.

To define whether preincubation with HSP impairs the antimicrobial activity of human neutrophils, neutrophils were challenged with uropathogenic *Escherichia coli* (UPEC) at MOIs of 10 and 100, representing a moderate infectious challenge and a high-burden condition, respectively. Our group previously reported that UPEC triggers NET release in neutrophils and that NET formation plays an important role in extracellular bacterial elimination *in vitro* (38).

**Figure 2:**
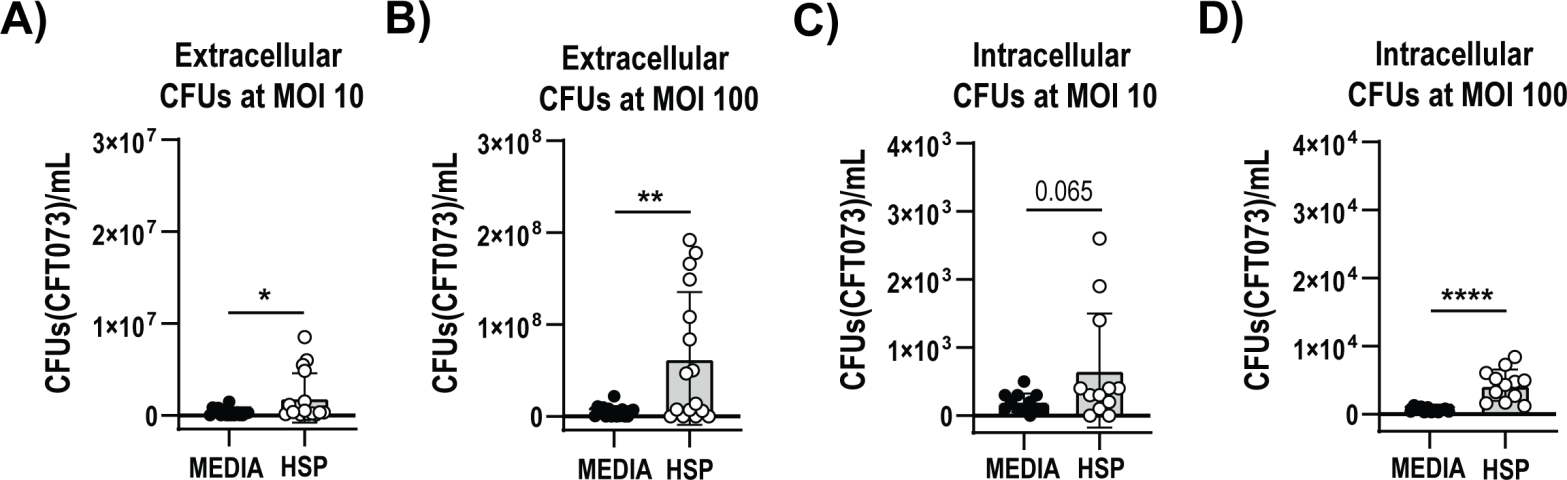
HSP impairs neutrophil-mediated killing of UPEC. **(A, B)** Bar graphs showing the extracellular colony-forming units (CFUs) in neutrophil cell cultures pre-incubated with HSP or media, and infected with UPEC at **(A)** MOI 10 and **(B)** MOI 100. **(C, D)** Bar graphs illustrate the intracellular CFUs of neutrophils pre-incubated with media or HSP and then stimulated with UPEC at **(C)** MOI 10, and **(D)** MOI 100. All data are presented as mean ± SD. Data were obtained from 2 independent experiments, with *n* = 8–12 per group. Statistical significance was determined by unpaired t-test. p < 0.05*, p < 0.01**, and p < 0.0001****.

We found that preincubation with HSP significantly inhibited UPEC-induced NETosis (*p <* 0.0001; **Figures 1A, D**), which was associated with an increased extracellular bacterial burden (**Figures 2A, B**).

To exclude the possibility that reduced NET formation and impaired antimicrobial activity in HSP-treated neutrophils was due to loss of cell viability, neutrophil cell death was determined by flow cytometry. Analysis of double-positive cells (7-AAD^+^/Annexin V^+^) cells revealed that stimulation with A23187, PMA, or UPEC induced approximately 20% cell death. In contrast, HSP alone did not impair neutrophil viability (0.83% dead cells), with a percentage of dead cells comparable to that of unstimulated neutrophils (0.34%, **Supplementary Figures 1A, B**).

Taken together, these data identify HSP as a negative regulator of NETosis and antibacterial function in neutrophils.

### 3.2 HSP reduces ROS generation in neutrophils stimulated with A23187, PMA, and UPEC

**Figure 3:**
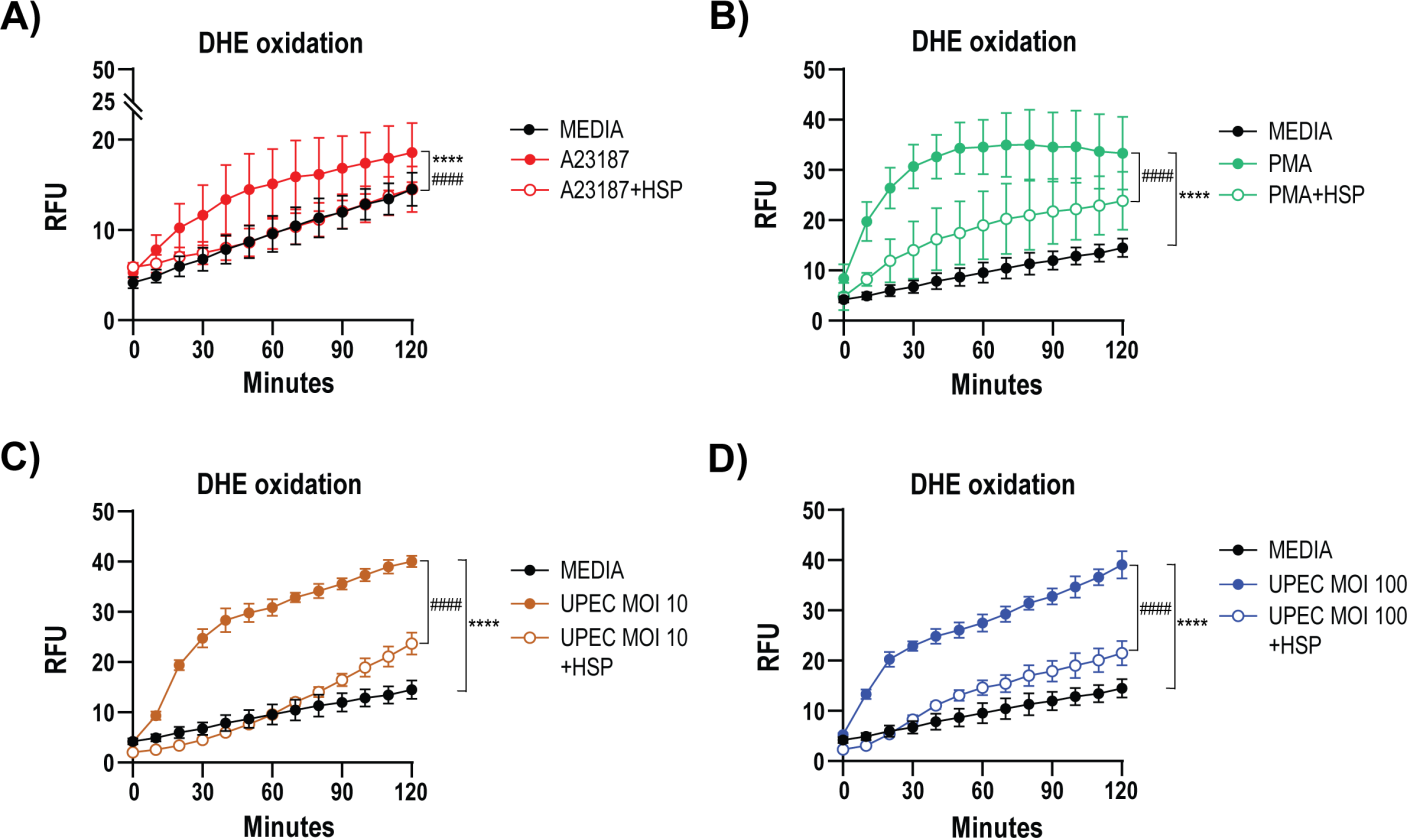
HSP reduces ROS production in human neutrophils stimulated with PMA, A23187, and UPEC. Graphs showing relative fluorescence units (RFU) of dihydroethidium (DHE) oxidation in human neutrophils preincubated with media or HSP, followed by stimulation for two hours with **(A)** A23187, **(B)** PMA, or **(C)** UPEC (CFT073) MOI of 10, and **(D)** MOI of 100. All data are presented as mean ± SD. Data were obtained from 2 independent experiments, with *n* = 8–12 per group. DHE oxidation was quantified by calculating the area under the curve (AUC) from kinetics fluorescence measurements. Statistical analysis was performed using ordinary one-way ANOVA followed by Tukey’s multiple comparisons test. **** Indicates a statistically significant difference compared with the media control group; ### indicates a statistically significant difference between the stimulus-only group and the stimulus + HSP group.

Given that A23187, PMA, and UPEC are potent inducers of ROS, we next evaluated the effect of HSP on ROS production by human neutrophils stimulated with these agents. We found that A23187 (**Figure 3A**), PMA (**Figure 3B**), and UPEC (**Figures 3C, D**) induced significantly elevated ROS levels, as measured by dihydroethidium (DHE) oxidation, in human neutrophils compared to media-treated controls. In contrast, neutrophils pre-incubated with HSP exhibited reduced ROS levels in response to A23187, PMA, and UPEC (**Figures 3A–D**). Given that previous studies from our laboratory have demonstrated a critical role for neutrophil ROS in the intracellular eradication of UPEC (38), we next quantified the intracellular bacterial load. HSP-treated neutrophils showed a marked increase in intracellular UPEC burden compared to infected media-treated cells (**Figures 2C, D**). Together, these findings indicate that HSP suppresses the oxidative burst in activated human neutrophils, an effect that is associated with impaired neutrophil-mediated UPEC elimination.

### 3.3 HSP impairs the phagocytic capacity of human neutrophils

**Figure 4:**
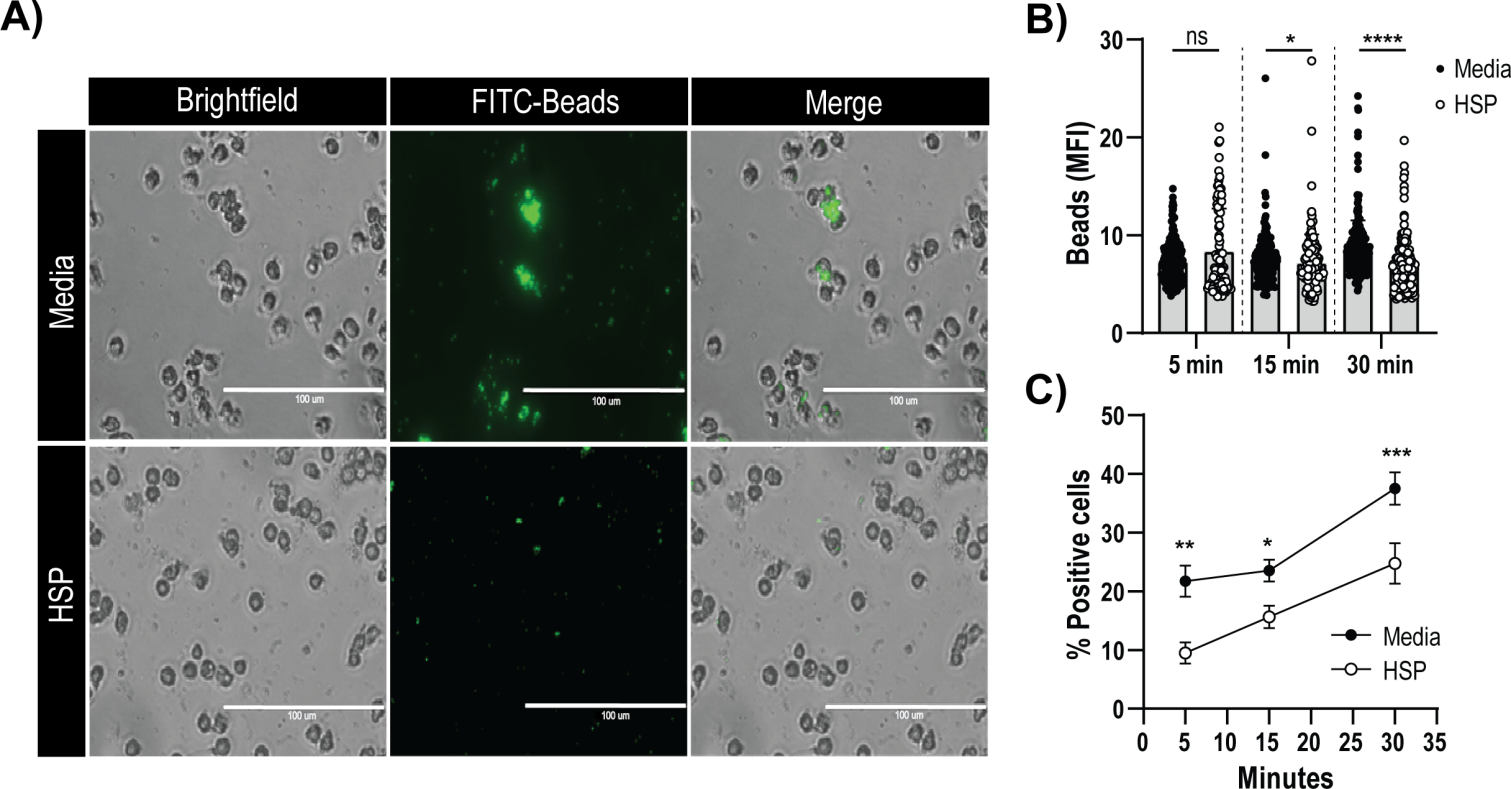
HSP affects the phagocytic uptake of FITC-LPS bioparticles by human neutrophils. **(A)** Representative figure of human neutrophils pre-incubated with media or HSP and then incubated with LPS-coated FITC microbeads for 30 minutes. Scale bar = 100 µm. **(B)** Bar graph showing the mean fluorescence intensity (MFI) of FITC-LPS bioparticles internalized by neutrophils at 5, 15, and 30 minutes. **(C)** Graph illustrates the percentage of neutrophils that phagocytosed FITC-LPS bioparticles at 5, 15, and 30 minutes. All data are presented as mean ± SD. Data were obtained from 2 independent experiments. **(B)** Statistical significance was determined using unpaired t-test. **(C)** Statistical significance was determined by two-way ANOVA followed by Šídák’s multiple comparisons test. p < 0.05*, p < 0.01**, p < 0.001***, and p < 0.0001****.

To determine whether HSP modulates additional effector functions in human neutrophils, we evaluated the phagocytic capacity of neutrophils pre-incubated with HSP and then exposed to fluorescein-labeled, LPS-coated bioparticles. Phagocytosis was quantified by measuring the MFI of internalized bioparticles over time. HSP-treated neutrophils displayed significantly lower MFI values than media-treated control cells at both 15 min (*p* = 0.0429) and 30 min (*p* < 0.0001) after exposure to FITC-LPS bioparticles (**Figures 4A, B**). In agreement with this result, the percentage of neutrophils that internalized FITC-LPS bioparticles was also reduced in the HSP-treated group (**Figure 4C**). Together, these findings indicate that HSP impairs neutrophil phagocytic function by decreasing both the extent of particle uptake and the proportion of phagocytic cells.

### 3.4 HSP suppresses NET formation via PAD4 but not NOX2

To gain mechanistic insight into how HSP modulates NETosis, we examined major signaling pathways involved in NET formation. NETosis can be triggered either by NOX2-dependent ROS production or by PAD4-mediated histone citrullination and chromatin decondensation downstream of calcium influx (20, 46, 54–56). To assess whether HSP regulates NETosis through NOX2 or PAD4, neutrophils isolated from wild-type (WT), NOX2 knockout (*Cybb-/-*), and PAD4-deficient (*Padi4-/-*) mice were treated with HSP or media, prior to stimulation with A23187.

**Figure 5:**
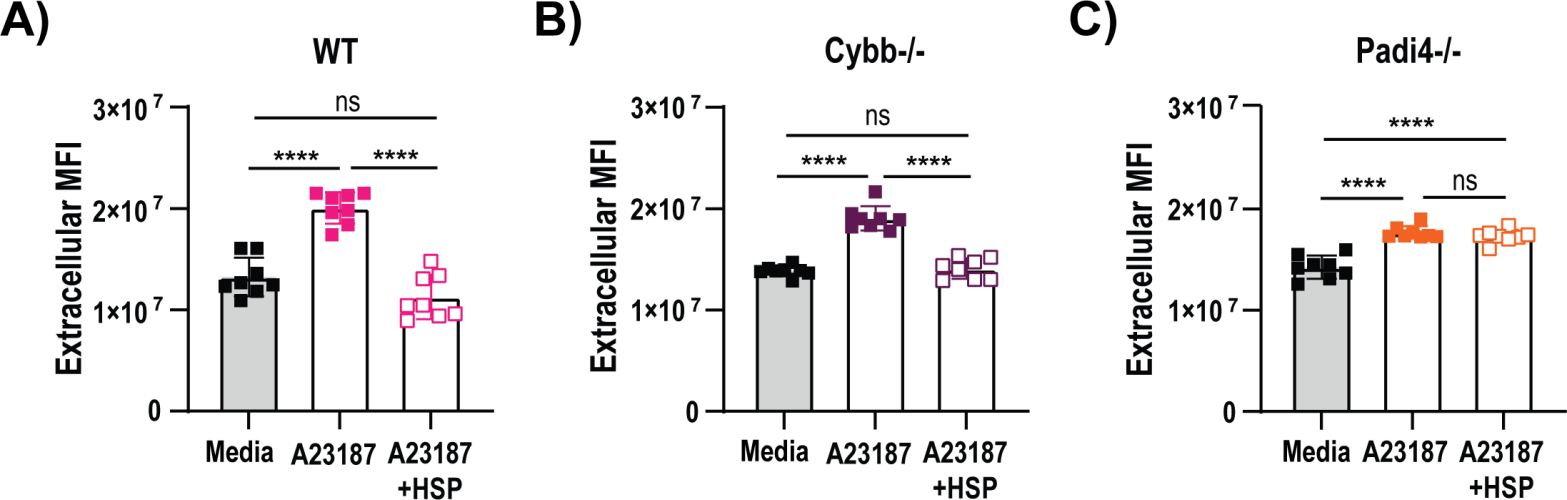
HSP inhibits A23187-induced NETosis in murine neutrophils in a PAD4-dependent but NOX2-independent manner. **(A-C)** Bar graphs showing the mean fluorescence intensity (MFI) of extracellular DNA release from **(A)** WT, **(B)** *Cybb-/-*, **(C)** *Padi4-/-* neutrophils undergoing NETosis following exposure to Media, A23187, and A23187 + HSP. All data are presented as mean ± SD. Data were obtained from 2 independent experiments, with *n* = 6–8 per group. Statistical significance was determined by one-way ANOVA followed by Tukey’s multiple-comparisons test. p < 0.001*** and p < 0.0001****.

NET formation was significantly reduced in WT murine neutrophils treated with HSP prior to stimulation with A23187 (**Figure 5A**). These findings suggest that the immunomodulatory effect of HSP on neutrophil NETosis is conserved across these species.

Furthermore, *Cybb-/-* neutrophils exhibited NET formation comparable to WT neutrophils following A23187 stimulation (**Supplementary Figure 2A**; *p* = 0.186), consistent with previous reports showing that A23187 induces NETosis primarily through a calcium–PAD4-dependent pathway (57).

Importantly, A23187-induced NETosis in *Cybb-/-* cells was effectively suppressed by HSP treatment (**Figure 5B**), indicating that the inhibitory effects of HSP on NET formation are mediated through a NOX2-independent mechanism.

Notably, *Padi4-/-* neutrophils remained capable of forming NETs following A23187 stimulation, although to a lower extent than WT neutrophils (**Supplementary Figure 2B**; *p* = 0.0019). However, HSP failed to substantially inhibit NET formation in *Padi4-/-* cells (**Figure 5C**).

Altogether, these findings indicate that PAD4 is required for HSP-mediated suppression of NETosis in murine neutrophils.

### 3.5 Prostaglandin signaling contributes to HSP-mediated inhibition of neutrophil ROS production

We next sought to define the mechanism by which HSP regulates ROS production in activated neutrophils. Previous studies have shown that prostaglandins (PGs) can inhibit ROS generation in activated human neutrophils (58). Because PGs are abundant in HSP (59), we investigated whether HSP-mediated inhibitory effect on neutrophil ROS production is dependent on PG signaling.

To address this, primary human neutrophils were pre-incubated with vehicle or a cocktail of prostaglandin receptor antagonists (PGs inhibitor), including antagonists of the prostacyclin (IP) receptor and the prostaglandin E_2_ (PGE_2_) receptor (EP2 and EP4). Neutrophils were then stimulated with PMA.

**Figure 6:**
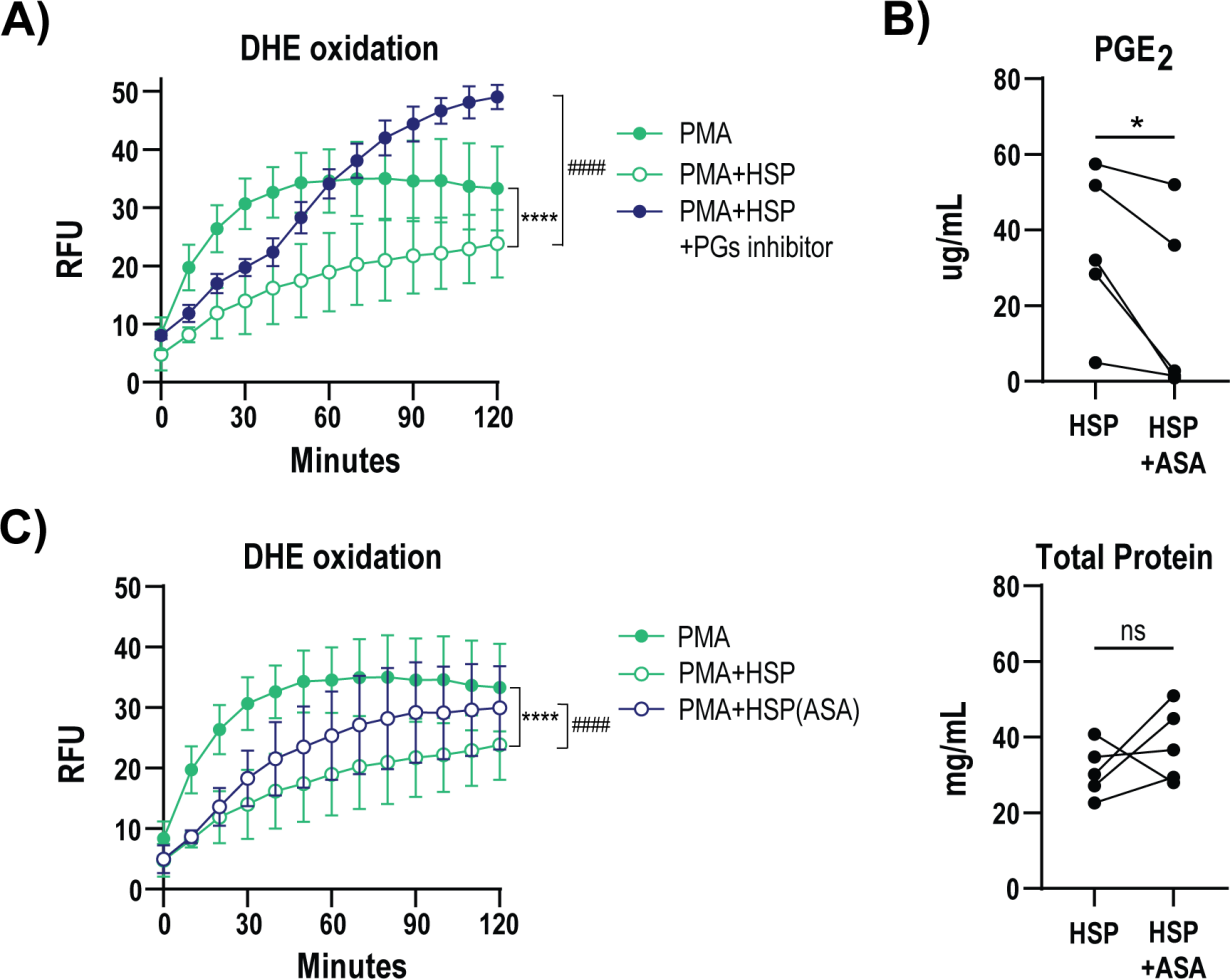
Prostaglandin signaling contributes to HSP-mediated inhibition of ROS production in PMA-stimulated neutrophils. **(A)** Kinetic graph showing RFU of DHE oxidation in human neutrophils preincubated with HSP in the presence or absence of prostaglandin receptor inhibitor cocktail (1 µM each inhibitor, 30 minutes), followed by PMA stimulation for two hours. **(B)** Quantification of PGE_2_ levels and total protein in HSP samples collected from donors before and after ASA ingestion. **(C)** Kinetic graph showing RFU of DHE oxidation in human neutrophils preincubated with HSP from donors before and after acetylsalicylic acid ingestion (ASA; HSP+ASA) and subsequently stimulated with PMA for two hours. Neutrophils treated with PMA alone are used as positive controls. All data are presented as mean ± SD (*n* = 8 per group). **(A, C)** was quantified by calculating the area under the curve (AUC) and analyzed by one-way ANOVA followed by Tukey’s multiple comparisons test. p < 0.001^###^ and p < 0.0001****. **(B)** was analyzed using paired t-test; * p < 0.05.

Following PMA stimulation, neutrophils exposed to HSP in the presence of prostaglandin receptor antagonists produced significantly higher levels of ROS than cells treated with HSP alone or vehicle (media) (**Figure 6A**). These findings indicate that prostaglandin signaling contributes to the suppressive effect of HSP on neutrophil ROS production.

To further substantiate these findings, neutrophils were pre-incubated with HSP obtained from donors treated with acetylsalicylic acid (ASA; 500 mg orally every 8 h for 3 days), a regimen known to inhibit cyclooxygenase (COX) activity and reduce PG synthesis (60–62). We corroborated that HSP from donors receiving ASA exhibited reduced PGE_2_ levels compared to HSP collected from untreated donors, while total protein remained unchanged (**Figure 6B**).

Under these conditions, HSP from ASA-treated donors was significantly less effective at suppressing PMA-induced ROS production than HSP from untreated donors (**Figure 6C**). Our findings suggest that prostaglandins in HSP contribute to negatively regulating ROS production in activated human neutrophils.

## 4 Discussion

In this study, we demonstrate that HSP exerts potent immunomodulatory effects on neutrophil effector functions. HSP suppressed both ROS production and NET formation, two mechanisms that are critical for pathogen containment and killing, in response to distinct physiologically relevant stimuli, including pharmacological agonists and bacteria (UPEC). This is in agreement with a recent report showing that seminal plasma inhibits NETosis in swine neutrophil–spermatozoa co-cultures, suggesting that this phenomenon is evolutionarily conserved across species and may not be restricted to humans (37).

The immunomodulatory role of HSP on neutrophils was accompanied by impaired UPEC clearance, highlighting a functional consequence of HSP-mediated neutrophil regulation. In women, sexual intercourse is a well-established risk factor for urinary tract infections (UTIs), the majority of which are caused by UPEC (63). Whether the inhibitory effects of HSP contribute to increased susceptibility to UTI following mating remains unknown and warrants further investigation.

The inhibitory effects of HSP on neutrophil antimicrobial functions may also have implications for susceptibility to sexually transmitted infections (STI), as neutrophils play a key role in mucosal defense against sexually transmitted pathogens (64, 65). However, the influence of seminal plasma on infection outcomes appears to depend on both the pathogen and the cell type involved. For example, seminal plasma has been shown to reduce *Chlamydia trachomatis* infection in endocervical epithelial cells, suggesting that its effects may differentially shape host defense mechanisms depending on the biological context.

Furthermore, our data show that inhibiting prostaglandin signaling via EP2/EP4 antagonism or reducing prostaglandin levels in HSP through donor ASA treatment attenuates HSP-mediated suppression of ROS production in activated neutrophils. These findings support our recently published hypothesis that prostaglandin-dependent signaling contributes to the ability of HSP to suppress neutrophil responses and may lead to impaired neutrophil antimicrobial functions (66). Consistent with this interpretation, previous studies have shown that seminal plasma contains high concentrations of prostaglandins with well-established immunomodulatory effects on inflammation and leukocyte function (67). In particular, PGE_2_ inhibits ROS production and NET formation in human neutrophils through activation of EP2 and EP4 receptors (54) (68). Nevertheless, the specific contribution of PGE_2_ to HSP-mediated neutrophil suppression remains unclear and warrants further investigation.

Beyond its effects on ROS production, our data further show that HSP suppresses NET formation through a PAD4-dependent, but NOX2-independent, mechanism. This observation is particularly relevant because it indicates that HSP does not merely inhibit neutrophils broadly but instead selectively modulates specific molecular pathways that govern NETosis (46, 54–56). The requirement for PAD4 suggests that HSP may interfere with calcium-dependent chromatin remodeling processes that are critical for NET release (46, 55). This selective regulation reinforces the idea that seminal plasma exerts a finely tuned influence on neutrophil effector functions (69, 70).

Although HSP-mediated immunosuppressive effects could potentially favor microbial persistence, they must be understood in the physiological context of reproduction. Seminal plasma is essential for the establishment of immune tolerance in the female reproductive tract, a process that supports sperm survival, fertilization, and early embryo development (69, 70). From an evolutionary perspective, mechanisms that limit neutrophil activation, ROS production, and NET formation following insemination may have been favored because they reduce immune response to sperm and paternal antigens, increasing reproductive success.

Ongoing work in our laboratory is addressing whether HSP can also exert systemic immunomodulatory effects on circulating neutrophils, with potential consequences for antimicrobial defense beyond the reproductive tract. More broadly, the significance of HSP-mediated regulation of NETosis may extend beyond infection. Excessive or dysregulated NET formation has been implicated in pregnancy complications such as preeclampsia, as well as in autoimmune and thromboinflammatory diseases (71, 72).

Overall, our findings identify HSP as a key regulator of neutrophil antimicrobial function and highlight its role in balancing reproductive immune tolerance and host defense, with potential consequences for bacterial survival and reproductive success.

## 5 Limitations

We would like to highlight the following limitations of our study. First, although the effects of HSP on neutrophil effector functions were demonstrated in primary human neutrophils, the mechanistic analysis of NOX2 and PAD4 dependence relied on murine knockout cells. Because murine and human neutrophils differ in several functional and phenotypic features, and because the use of HSP in murine neutrophil experiments may introduce interspecies differences that are not fully representative of HSP–human neutrophil interactions, the mechanistic interpretation of these findings should be extrapolated to human biology with caution. However, the observation that HSP also suppressed NETosis in WT murine neutrophils suggests that at least some of its immunomodulatory effects may be conserved across species.

Second, although sufficient to detect *in vitro* effects, the sample size may not fully capture interindividual variability in HSP composition or neutrophil responsiveness. Moreover, the limited number of HSP donors analyzed may not represent the full biological variability of seminal plasma, warranting validation in larger donor cohorts.

Third, bacterial killing assays were performed using a single reference UPEC strain, CFT073. Given the marked genetic and virulence heterogeneity among UPEC isolates, the impact of HSP on bacterial clearance may vary across strains. Therefore, our findings should not be generalized to all UPEC variants without validation in additional clinical isolates.

Fourth, all experiments were conducted *in vitro* using isolated neutrophils. Although this approach allowed precise assessment of neutrophil-intrinsic responses, it does not fully reproduce the complex microenvironment of the female genitourinary tract, where pH, epithelial barriers, microbiota, hormonal signals, and immune cell crosstalk are likely to influence neutrophil function. In addition, our study examined acute exposure to HSP, whereas repeated *in vivo* exposure may induce additional regulatory effects not captured by the present model.

Fifth, prostaglandin signaling was implicated as a factor contributing to the regulatory activity of HSP based on pharmacological inhibition and the use of HSP from aspirin-treated donor. However, the precise biochemical mediators responsible for the observed effects remain to be fully defined. Additionally, HSP was tested at a single dilution, and dose–response studies were not performed. Therefore, whether the observed effects are concentration dependent remains to be determined.

## Data Availability

The original contributions presented in the study are included in the article/**Supplementary Material**. Further inquiries can be directed to the corresponding authors.
