# Supplementary material for: Human seminal plasma suppresses neutrophil antimicrobial functions and promotes bacterial survival
Source: Front Immunol. 2026 Jul 14;17:1846703. doi: 10.3389/fimmu.2026.1846703 (PMC13407980; doi:10.3389/fimmu.2026.1846703)
Supplement: Supplementary file 1 [file Image1.pdf]

Supplementary Figures

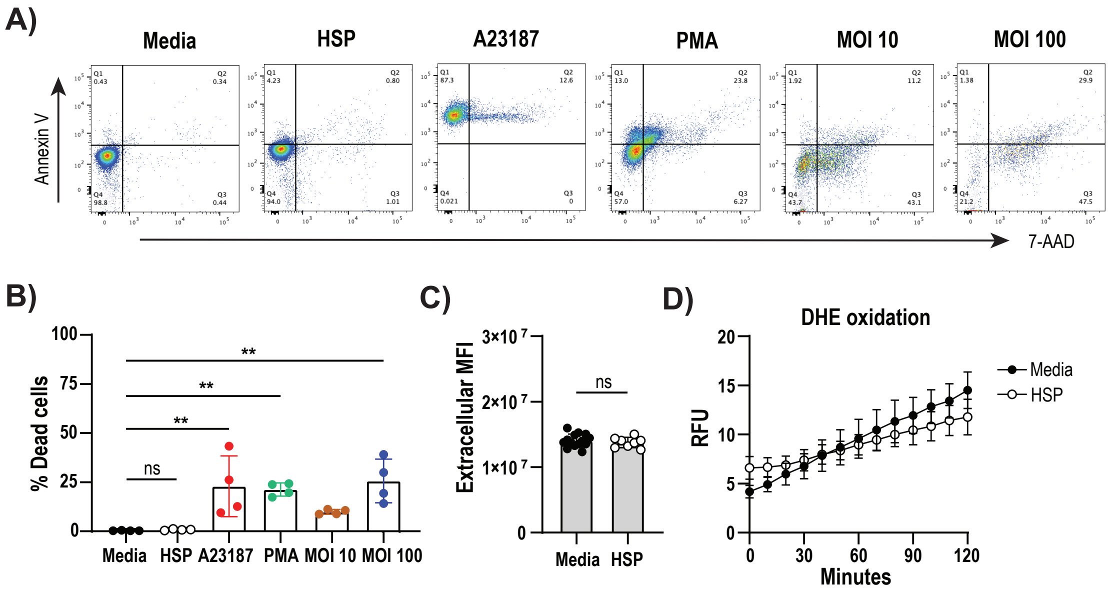

Supplemental Figure 1. (A) Representative flow cytometry plots and **(B)** bar graph showing the percentage of dead human neutrophils either untreated or pre-incubated with human seminal plasma (HSP), after stimulation with calcium ionophore A23187, phorbol 12-myristate 13-acetate (PMA), or uropathogenic *Escherichia coli* (UPEC, strain CFT073) at multiplicities of infection (MOIs) of 10 and 100. Double-positive 7-AAD+/Annexin V+ cells were considered dead cells. Data are shown as mean ± SD. $N = 4$ per group. Statistical significance was determined by ordinary one-way ANOVA followed by Dunnett's multiple comparisons test (**$p < 0.01$; ns, not significant). **(C)** Bar graph showing the mean fluorescence intensity (MFI) of extracellular DNA release in human PMNs, either untreated or pre-incubated with HSP. Data are presented as mean ± SD. $n = 8$ per group. **(D)** Graph showing relative fluorescence units (RFU) of dihydroethidium (DHE) oxidation in human PMNs, either untreated or pre-incubated with HSP. Data are presented as mean ± SD. $n = 10$ per group. DHE oxidation was quantified by calculating the area under the curve (AUC) from kinetics fluorescence measurements, these were analyzed with unpaired t-test.

.

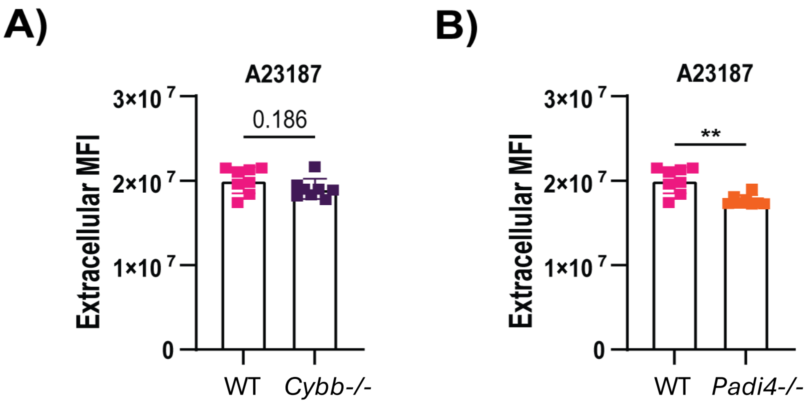

Supplemental Figure 2. (A–B) Quantification of extracellular DNA release, measured as mean fluorescence intensity (MFI), in murine neutrophils stimulated with A23187. **(A)** WT and *Cybb-/-* neutrophils. **(B)** WT and *Padi4-/-* neutrophils. Data are presented as mean ± SD and were pooled from two independent experiments (**n** = 6–8 per group). Statistical significance was assessed using an unpaired two-tailed *t*-test. **P** < 0.01 (**).
